# Utilization of Computed Tomography Colonography (CTC) at a District General Hospital: A Retrospective Analysis

**DOI:** 10.7759/cureus.84490

**Published:** 2025-05-20

**Authors:** Ali Javaid, Kehkashan Anwar, Ehtisham Zeb, Harry Large, Nafees Qureshi

**Affiliations:** 1 General and Colorectal Surgery, Tameside and Glossop Integrated Care NHS Foundation Trust, Manchester, GBR; 2 General Surgery, Tameside and Glossop Integrated Care NHS Foundation Trust, Manchester, GBR

**Keywords:** colonic pathology, colonic screening, colonoscopy, colorectal cancer, ctc, ctvc

## Abstract

Colorectal cancer (CRC) is one of the most common cancers worldwide, necessitating efficient diagnostic pathways. While colonoscopy remains the gold standard, computed tomography colonography (CTC) offers a non-invasive alternative, particularly for elderly and comorbid patients. This retrospective study, conducted at a UK district general hospital (DGH), evaluated patterns of CTC utilization among patients referred for colonic investigations over a 12-month period. Despite CTC’s high diagnostic accuracy (93% sensitivity for polyps > 10 mm) and its suitability for high-risk populations, it was used as a first-line investigation in only 3.5% of cases, markedly lower than the 12%-15% reported in tertiary centers. Contributing factors included limited radiologist training, restricted CT scanner capacity, and a prevailing institutional preference for colonoscopy. Addressing these DGH-specific barriers, such as resource constraints and workforce limitations, is critical to enhancing CTC’s role within the two-week-wait (2WW) pathway. This study underscores the need for multicenter research to validate these findings and guide policy development, ensuring equitable access to non-invasive diagnostics for frail, comorbid, or preference-driven populations.

## Introduction

Colorectal cancer (CRC) remains the third most common cancer globally, necessitating efficient and cost-effective diagnostic pathways [1]. Colonoscopy is the gold standard for direct visualization and biopsy of suspected lesions; however, computed tomography colonography (CTC) has emerged as a non-invasive alternative, particularly for elderly or comorbid patients [2]. The UK's two-week-wait (2WW) pathway prioritizes rapid cancer diagnosis, but challenges such as incomplete colonoscopies and patient frailty complicate adherence [3].

CTC offers several advantages, including the absence of sedation, a lower risk of perforation (<0.03% vs. 0.1% for colonoscopy), and effectiveness in cases involving obstructing lesions [4]. Systematic reviews suggest that, when performed according to best practice by experienced practitioners, CTC and colonoscopy offer comparable sensitivity for detecting CRC and polyps [5-8].

Despite guideline recommendations supporting CTC for incomplete colonoscopy or high-risk patients, its adoption in district general hospitals (DGHs) remains inconsistent [9]. 

Aims and objectives

This study evaluates CTC utilization patterns in a UK DGH, focusing on clinical indications, patient demographics, and implementation challenges associated with its use as a diagnostic modality for colorectal pathologies. It also compares local utilization rates with those reported in tertiary centers. The findings highlight the need for multicenter studies to inform policy development and standardize diagnostic pathways across diverse hospital settings.

## Materials and methods

This retrospective observational study, conducted at Tameside and Glossop Integrated Care NHS Foundation Trust, Fountain Street, Ashton-under-Lyne, England, analyzed all patients referred for CTC between January and December 2022. Data were collected using three primary electronic systems: Lorenzo (electronic patient records), Unisoft (GI reporting tool), and Sectra PACS Uniview (radiology image and reporting system). The study was conducted in accordance with institutional ethical standards and the Declaration of Helsinki. As it involved a retrospective review of anonymized patient data, formal ethical approval was not required.

Inclusion criteria

All patients referred for CTC during the defined study period, regardless of indication, were included in the analysis.

Exclusion criteria

No exclusion criteria were applied, ensuring comprehensive data capture for all referrals within the calendar year.

CTC technique

The CTC technique involved oral Gastrografin preparation for tagging, IV administration of 20 mg Buscopan, rectal insufflation using a CO₂ auto-insufflator, and image acquisition in both supine and prone positions. IV contrast was not routinely used. CTC studies were reported either by consultant radiologists with a gastrointestinal (GI) interest or by trained advanced radiography practitioners under consultant supervision.

Variables collected

Data points extracted included age (in years), sex (male/female), referral indication (e.g., frailty, patient preference, and failed colonoscopy), and the clinical rationale for CTC use (first-line vs. post-colonoscopy).

Statistical analysis

All statistical analyses were performed using IBM SPSS Statistics for Windows, Version 28.0 (Released 2021; IBM Corp., Armonk, NY, US). Descriptive statistics were calculated, including means and standard deviations (mean ± SD) for continuous variables (e.g., age), and counts and percentages (N, %) for categorical variables (e.g., sex, referral reasons). Group comparisons for categorical variables were conducted using chi-square (χ²) tests to assess differences between groups (e.g., first-line vs. second-line CTC). A p-value < 0.05 was considered statistically significant.

## Results

The study included 389 patients who underwent CTC, comprising 63.3% women (n = 246) and 36.7% men (n = 143). Patient ages ranged from 28 to 98 years, with a mean age of 68.2 years (SD ± 12.4) (Table 1).

**Table 1:** Demographic characteristics of CTC patients (N = 389) Gender data is represented as N (%) and age as mean ± SD. No statistical test was applied. CTC:  computed tomography colonography.

Characteristic	N	%	Value
Female	246	63.3%	—
Male	143	36.7%	—
Age (mean ± SD)	—	—	68.2 ± 12.4

Gender distribution among the CTC subgroups did not show any significant differences (Table 2). However, there was a statistically significant difference in age between these subgroups (Table 3).

**Table 2:** Gender distribution by CTC type χ² = 0.81. p = 0.3683. Data represented as N (%). Statistical test: chi-square test of independence. Significance threshold set at p < 0.05. CTC: computed tomography colonography.

CTC type	Female (N, %)	Male (N, %)	Total
First-line CTC	100 (62.9%)	59 (37.1%)	159
Second-line CTC	156 (67.8%)	74 (32.2%)	230

**Table 3:** Age comparison between first-line and second-line CTC t-value = 4.69. p < 0.001. Age data represented as mean ± SD. Statistical test: independent samples t-test. Significance threshold set at p < 0.05, with p < 0.001 considered highly significant. CTC: computed tomography colonography.

CTC type	Mean age ± SD	N
First-line CTC	70.0 ± 12.0 years	159
Second-line CTC	67.0 ± 13.0 years	230

**Figure 1:**
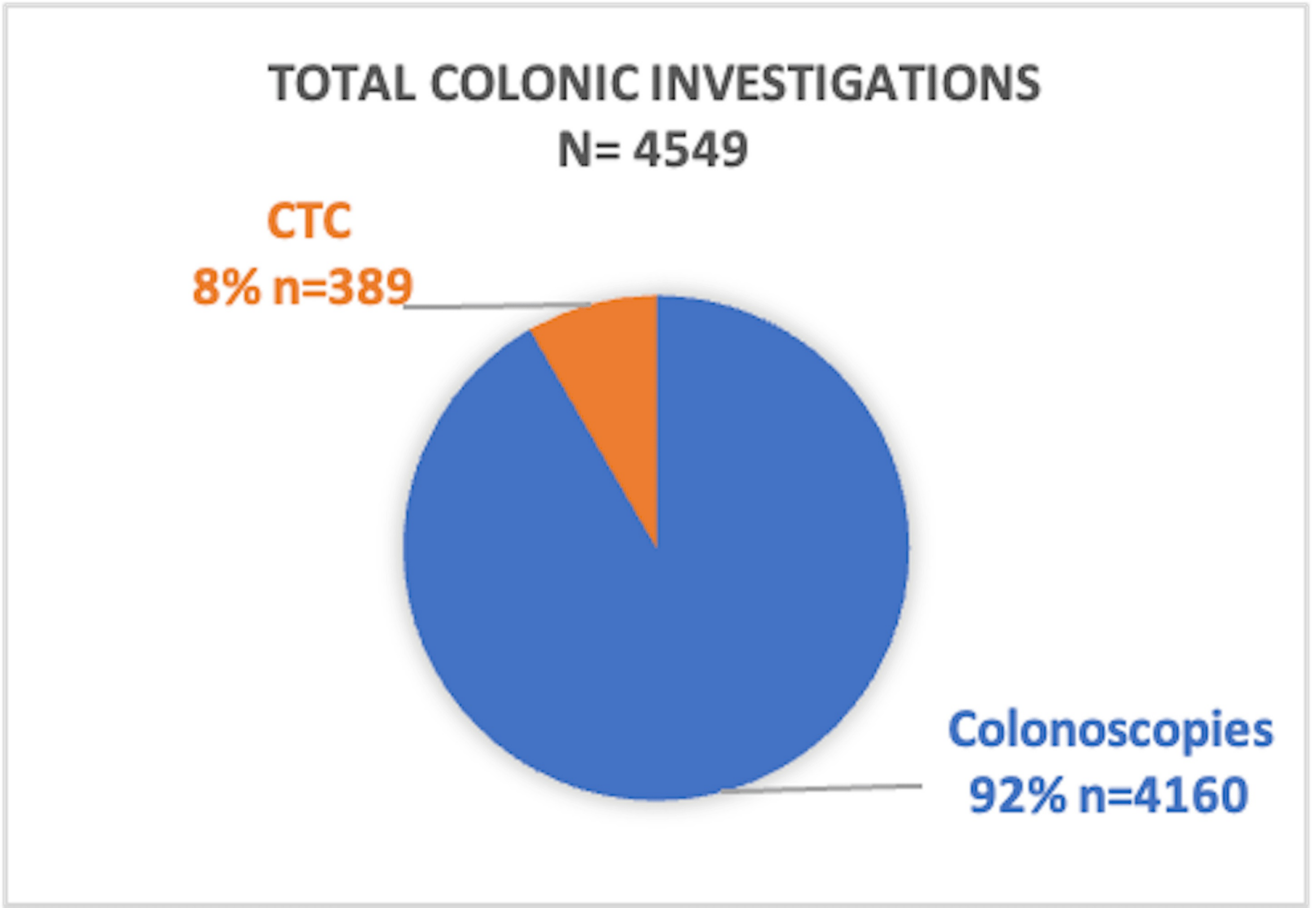
Distribution of colonic investigations (N = 4549)

Among 4549 colonic investigations performed, 4160 were colonoscopies and 389 were CTCs, with CTC accounting for 8% (n = 389) of all procedures (Figure 1).

**Figure 2:**
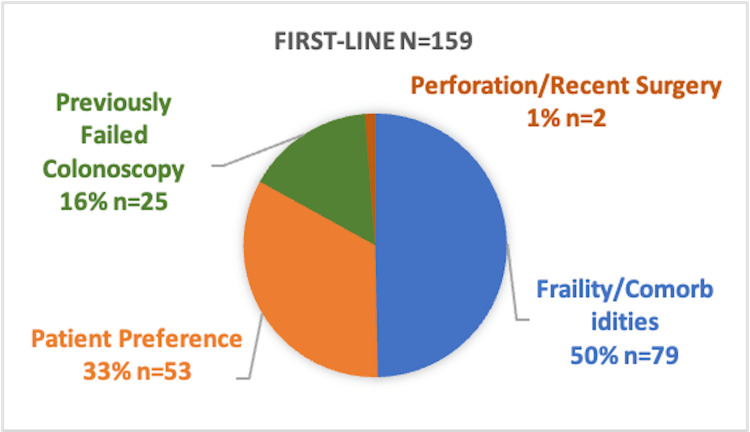
Indications for first-line CTC (N = 159) CTC: computed tomography colonography.

First-line CTC accounted for 41% (n = 159) of CTC cases, with the primary indications being frailty/comorbidities (50%, n = 79), patient preference (33%, n = 53), previously failed colonoscopy (16%, n = 25), and perforation history/recent surgery (1%, n = 2). The remaining 59% (n = 230) of CTCs were performed following failed or incomplete/inadequate colonoscopy (Figure 2).

Overall, first-line CTC accounted for only 3.5% (n = 159) of all colonic investigations, indicating its underutilization within this cohort.

Detailed statistical analyses are presented in Table 4.

**Table 4:** Summary of CTC utilization and indications in the study cohort (N = 4549) Data are presented as N (number), % (percentage), and mean ± SD (standard deviation) as appropriate. Comparisons between groups were analyzed using chi-square (χ²) tests for categorical variables. Where applicable, the corresponding test statistic (χ² value) has been reported. A p < 0.05 was considered statistically significant. CTC: computed tomography colonography.

Parameter	N	%	Comparison group	Test statistic (χ²/t/F)	p-value
Total colonic investigations	4549	100%	—	—	—
Colonoscopies performed	4160	92%	CTC vs. colonoscopy	χ² = 2852.7	<0.001
CTCs performed	389	8%	CTC vs. colonoscopy	—	—
First-line CTC	159	3.5%	First-line vs. all CTCs	χ² = 19.0	<0.001
Second-line CTC (post-unsuccessful colonoscopy)	230	59.1%	First-line vs. second-line	χ² = 19.0	<0.001
Indications for first-line CTC (n = 159)					
Frailty/comorbidities	79	50%	Frailty vs. others	χ² = 56.8	<0.001
Patient preference	53	33%	Preference vs. others	χ² = 32.0	<0.001
Previously failed colonoscopy	25	16%	Previously failed vs. others	χ² = 7.5	0.006
Recent surgery/perforation history	2	1%	Surgery vs. others	χ² = 144.9	<0.001
Age of patients undergoing CTC (n = 389)	—	—	Mean ± SD	—	—
Age (years)	—	—	68.2 ± 12.4	—	—
Sex distribution (CTC patients, n = 389)			Female vs. male	χ² = 29.8	<0.001
Female	246	63.3%			
Male	143	36.7%			

## Discussion

CTC is a highly sensitive and well-tolerated test for diagnosing colorectal pathologies [10]. According to the UK government bowel cancer screening report, 64,440 people underwent a CTC scan as part of the NHS bowel cancer screening program in 2021-2022. On average, over 100,000 CTCs are performed annually in the UK, with this number increasing each year [11]. When conducted according to best practice by experienced practitioners, CTC demonstrates excellent diagnostic accuracy for colorectal neoplasms in both symptomatic and screening populations [2,12-15].

Underutilization of CTC

Despite evidence supporting CTC’s high diagnostic accuracy (93% sensitivity for polyps > 10 mm) [12], it accounted for less than 10% of colonic investigations in this cohort. In comparison, a 2020 UK audit reported CTC utilization rates of 12%-15% in tertiary centers [16], highlighting the significant challenges DGHs face in matching these figures. Capacity constraints and insufficient training in CTC interpretation are among the most common barriers [17].

First-line CTC indications

Frailty and comorbidities account for nearly 50% (n = 79) of first-line referrals, consistent with studies advocating CTC for high-risk elderly patients [18]. Patient preference (33%, n = 53) reflects the growing acceptance of non-invasive diagnostics, although colonoscopy remains culturally entrenched [19].

Post-colonoscopy CTC

Most referrals (59%, n = 230) followed incomplete colonoscopy, consistent with the ESGE guidelines [20].

Emerging indications

Emerging evidence supports the use of CTC as a first-line investigation in patients with sigmoid colostomy and known deep pelvic endometriosis [21,22]. CTC can also aid in differentiating between inflammatory and neoplastic strictures in patients with extensive diverticular disease [23-25]. The role of CTC as a first-line screening tool for CRC remains under debate. Currently, in the UK, its use is limited to populations deemed unfit for colonoscopy. European studies suggest greater public acceptance of CTC compared to colonoscopy [26,27]. Notably, increased participation in screening programs was observed with CTC versus colonoscopy (34% vs. 22%; p < 0.0001) in the Dutch trial [15].

The Greater Manchester Lower GI Triage and Colonic Imaging Guidelines recommend CTC as a first-line investigation in patients classified as low risk for CRC based on fecal immunochemical test (FIT) stratification [28].

Limitations

The single-center design limits the generalizability of the findings. As a retrospective study, the data may underreport nuanced clinical decisions. Additionally, the definitions of frailty and severity of comorbidities were not standardized and depended on individual clinicians' assessment.

Future directions

Expand Radiologist Training Programs

CTC interpretation requires specialized expertise in analyzing 3D images to accurately detect polyps and CRCs while differentiating them from stool residues and anatomical folds. To address this, establishing structured mentorship programs with experienced CTC radiologists is essential. Furthermore, a formal certification program administered by the Royal College of Radiologists can standardize training and enhance proficiency. These measures will help increase the number of radiologists skilled in CTC, thereby improving diagnostic capacity and quality.

Improve the Capacity of the Radiology Department to Perform the Procedure

This can be achieved by improving equipment, such as access to advanced CT scanners, availability of automated insufflators, and the latest 3D reconstruction software, and by increasing staffing levels through the recruitment and training of technicians and radiologists specialized in performing and reporting CTC.

## Conclusions

CTC is a valuable non-invasive diagnostic tool for colorectal conditions, demonstrating high sensitivity and specificity. Despite its clinical benefits, particularly for frail, comorbid, or preference-driven individuals, it remains underutilized in our DGH. Individual DGHs can address this by investing in additional trained technicians and radiologists to increase CTC capacity. At the national level, further multicenter studies are essential to inform policy development and standardize diagnostic pathways across the UK’s National Health Service. Additional research is also needed to explore CTC’s utility in emerging indications such as patients with sigmoid colostomy, pelvic endometriosis, screening populations, and low-risk individuals stratified by FIT.
